# Computation and application of tissue-specific gene set weights

**DOI:** 10.1093/bioinformatics/bty217

**Published:** 2018-04-06

**Authors:** H Robert Frost

**Affiliations:** Department of Biomedical Data Science, Geisel School of Medicine at Dartmouth, Hanover, NH, USA

## Abstract

**Motivation:**

Gene set testing, or pathway analysis, has become a critical tool for the analysis of high-dimensional genomic data. Although the function and activity of many genes and higher-level processes is tissue-specific, gene set testing is typically performed in a tissue agnostic fashion, which impacts statistical power and the interpretation and replication of results.

**Results:**

To address this challenge, we have developed a bioinformatics approach to compute tissue-specific weights for individual gene sets using information on tissue-specific gene activity from the Human Protein Atlas (HPA). We used this approach to create a public repository of tissue-specific gene set weights for 37 different human tissue types from the HPA and all collections in the Molecular Signatures Database. To demonstrate the validity and utility of these weights, we explored three different applications: the functional characterization of human tissues, multi-tissue analysis for systemic diseases and tissue-specific gene set testing.

**Availability and implementation:**

All data used in the reported analyses is publicly available. An R implementation of the method and tissue-specific weights for MSigDB gene set collections can be downloaded at http://www.dartmouth.edu/∼hrfrost/TissueSpecificGeneSets.

## 1 Introduction

Gene set testing, or pathway analysis, has become an indispensable tool for the analysis and interpretation of high dimensional genomic data, including measures of DNA sequence variation, DNA methylation, RNA expression and protein abundance (Hung *et al.*, 2012; Khatri *et al.*, 2012). By focusing on the collective effect of biologically meaningful groups of genomic variables, rather than just the marginal effect of individual variables, gene set testing methods can significantly improve statistical power, replication of results and biological interpretation (Allison *et al.*, 2006; Goeman and Buhlmann, 2007). Although significant progress has been made building gene set collections (Gene Ontology Consortium, 2010; Liberzon *et al.*, 2011) and developing statistical gene set testing methods (Subramanian *et al.*, 2005; Wu and Smyth, 2012), the practical utility of gene set testing has been limited, with major challenges including annotation quality, statistical power and tissue specificity.

### 1.1 Tissue-specificity of genes and processes

It is well known that the expression and function of many genes is strongly linked to tissue context (Bossi and Lehner, 2009; Dezso *et al.*, 2008; Ju *et al.*, 2013; Keshava Prasad *et al.*, 2009; Winter *et al.*, 2004) [e.g. coagulation factor II (thrombin) is enriched in the liver (Uhlén *et al.*, 2015)], with tissue-specificity extending to a sizable number of higher-level pathways, processes and cellular functions [e.g. keratinocyte differentiation is specific to the epidermis (Pierson *et al.*, 2015)]. Until recently, however, researchers have lacked a comprehensive and accurate understanding of the tissue specificity of all human protein-coding genes with repositories of gene-tissue mappings, e.g. Human Protein Resource Data Base (Keshava Prasad *et al.*, 2009) and BRENDA enzyme information system (Chang *et al.*, 2015), based largely on associations manually curated from the biomedical literature. A comprehensive view of tissue-specific gene activity is now beginning to emerge with the publication of results from projects such as the Human Protein Atlas (HPA; Uhlén *et al.*, 2015), ProteomicsDB (Wilhelm *et al.*, 2014), Human Proteome Map (Kim *et al.*, 2014) and Gene-Tissue Expression Project (GTEx; GTEx Consortium, 2015). Using techniques including immunohistochemistry (IHC), deep sequencing transcriptomics and mass spectrometry, these projects are investigating the tissue-specific activity of the ∼20 000 human protein-coding genes with early results clearly demonstrating the importance of tissue context. According to results from the HPA, approximately 34% of all human protein-coding genes have elevated expression in at least one tissue with 17% showing expression levels that are five times the maximum measured in any other tissue. Importantly, less than half of all protein-coding genes (approximately 44%) were found in the HPA analysis to be ubiquitously expressed in all tissue types (this includes 60% of the metabolic enzymes, a large proportion of transcription factors and many other ‘housekeeping’ genes). In addition to improved knowledge about the tissue-specific expression of genes, important progress has also been made modeling the relationships between human tissues, e.g. the BRENDA Tissue Ontology (Gremse *et al.*, 2011) defines the hierarchical relationships between the major human tissue types, and in characterizing tissue-specific gene relationships (Greene *et al.*, 2015; Pierson *et al.*, 2015), often represented as gene networks with a distinct network per tissue type.

### 1.2 Current support for tissue-specific gene set testing

Although significant effort has been expended characterizing the tissue-specific activity of human genes, little information currently exists regarding the tissue-specificity of gene sets or for leveraging that knowledge during gene set testing. The Gene Ontology (GO; Gene Ontology Consortium, 2010) does include limited information, via annotation extensions (Huntley *et al.*, 2014), regarding the tissue or cell type associated with an annotation, however, only a small number of GO annotations have such tissue type extensions and no general support or tools are available for leveraging these extensions (or other sources of knowledge regarding tissue-specificity) to create tissue-specific versions of GO. For other standard gene set collections, e.g. Molecular Signatures Database (MSigDB; Liberzon *et al.*, 2011), information regarding the tissue specificity of gene sets and gene set annotations is completely lacking. Although more work has been done at the level of entire gene sets, e.g. Pierson *et al.* (2015) used keyword searching to identify a subset of GO terms that represent tissue-specific functions or processes, general purpose tools that can be used to compute the tissue-specificity for any gene set collection for any human tissue type do not yet exist. Furthermore, no available gene set testing methods are able to leverage knowledge regarding tissue-specific gene relationships. Although the work of Pierson *et al.* does provide a basis for tissue-based filtering of GO terms, their effort was based on keyword searching rather than experimental evidence. Because tissue-specific versions of gene set collections are not available or easy to create, it is currently standard practice to perform gene set testing using the same, generic gene sets and annotations regardless of the experimental tissue type. This practice is even common for projects investigating the tissue-specificity of human genes, e.g. standard GO terms and annotations were used to analyze the tissue-specific gene networks in Greene *et al.* (2015), the gene co-expression networks in Pierson *et al.* (2015) and differentially expressed genes in Uhlén *et al.* (2015).

### 1.3 Impact of tissue-specificity on gene set testing

If the annotations for all tested gene sets were to ubiquitously expressed genes, the current practice of ignoring tissue specificity would have little impact on gene set testing accuracy. However, because a large proportion of human genes do display tissue-specific activity (Uhlén *et al.*, 2015), performing gene set testing without regard to the tissue-specific activity of genes can be expected to elevate both the type I and type II error rates, perhaps substantially. The problem is further exacerbated by the fact that the evidence supporting gene set annotations in collections such as GO (Gene Ontology Consortium, 2010) is often based on an experiment conducted in a single tissue, with the annotations for a single gene set sometimes drawn from multiple tissue sources. If gene sets are tested that represent processes which never occur in the experimental tissue under analysis, the multiple hypothesis correction (MHC) burden will simply be increased without any chance of finding additional true associations. Even when the tested gene set is relevant for the target tissue, if the set contains annotations based on evidence associated with tissues other than the tissue under analysis, the computed gene set statistic may be biased.

## 2 Materials and methods

**Fig. 1.**
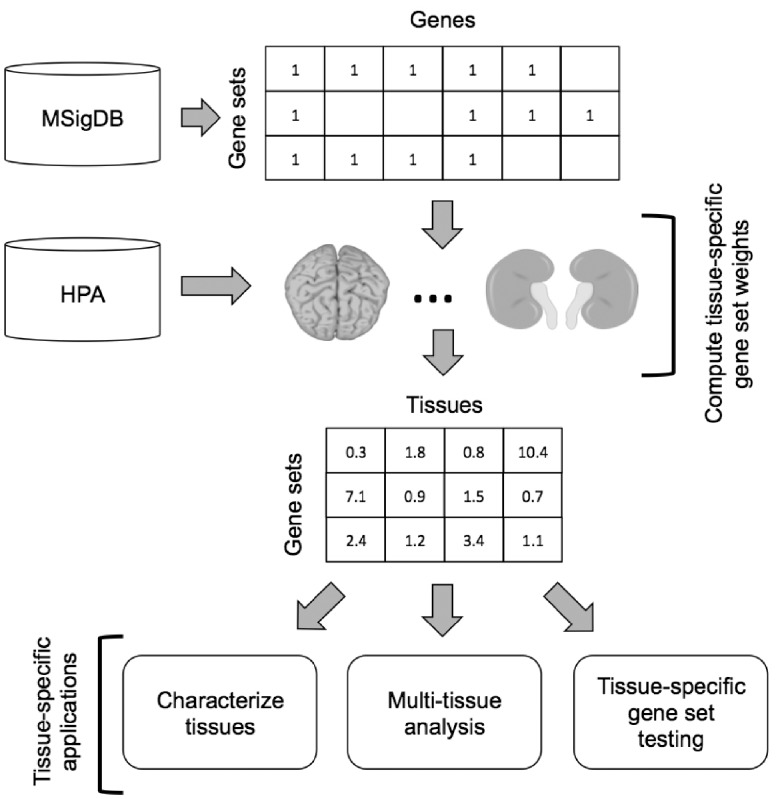
Conceptual representation of the proposed approach for computing and using tissue-specific gene set weights. The target gene set collection, e.g. one of the collections from the MSigDB, is represented as a matrix of indicator variables with rows representing gene sets, columns representing genes and elements set to 1 if an annotation exists between the corresponding gene and gene set. Using information from the HPA regarding gene activity in different human tissues, tissue-specific weights are computed according to the process detailed in Section 2.2 for all of the gene sets in the collection. Potential applications of these weights include the functional characterization of human tissues, tissue-specific gene set testing and multi-tissue analyses

To address the challenge of tissue-specificity for gene set testing, we have developed a new bioinformatics approach, illustrated in Figure 1, that uses information about tissue-specific gene function to compute a vector of weights for a given gene set (one weight per human tissue type) that can be leveraged during later analysis. The following sections outline the statistical details of the method and the leveraged data sources. Example applications of the computed tissue-specific gene set weights are detailed in Section 3.

### 2.1 Data sources

#### *2.1.1* Gene sets

The results described in this paper were based on gene sets from version 6.0 of the MSigDB (Liberzon *et al.*, 2011) as downloaded from http://software.broadinstitute.org/gsea/downloads.jsp. In particular, tissue-specific gene set weights were computed using the procedure detailed in Section 2.2 on 13 distinct MSigDB collections, as detailed in Table 1.

**Table 1.** Analyzed MSigDB gene set collections

ID	Collection name	# Sets
H	Hallmark gene sets	50
C1	Positional gene sets	326
C2.CGP	Chemical and genetic perturbations	3402
**C2.CP**	**Canonical pathways**	**1329**
C3.MIR	microRNA targets	212
C3.TFT	Transcription factor targets	615
C4.CGN	Cancer gene neighborhoods	427
C4.CM	Cancer modules	431
**C5.BP**	**GO biological process**	**4436**
C5.CC	GO cellular component	580
C5.MF	GO molecular function	901
C6	Oncogenic signatures	189
C7	Immunologic signatures	4872

*Note*: The 13 MSigDB version 6.0 collections for which tissue-specific gene set weights were computed. The collections marked in bold (C2.CP and C5.BP) were used to generate the analysis results in Section 3.

#### *2.1.2* Tissue-specific gene function

Information regarding the tissue-specificity of human protein-coding genes was drawn from version 16 of the HPA (Uhlén *et al.*, 2015) as downloaded from http://www.proteinatlas.org/about/download. Evidence for the tissue-specificity of genes was based on both HPA mRNA expression data as computed via RNA-seq and HPA protein abundance data as computed via IHC. See Section 2.2.1 below for more details.

### 2.2 Analysis pipeline

The analysis pipeline illustrated in Figure 2 takes as input a human tissue type *t* drawn from the set of tissue types supported by the HPA and a gene set collection represented by a *g *×* p* indicator matrix **G** that holds *g* gene sets annotated to *p* genes. The pipeline uses these inputs to compute tissue-specific gene set weights using the following steps (see Sections 2.2.1 and 2.2.2 below for more details on each step):

- **Assign tissue-specific gene weights:** For all genes annotated to the gene sets in **G**, a set of tissue-specific weights are computed according to the activity of the gene in the tissue types supported by the HPA.
- **Compute tissue-specific gene set weights:** The gene-level weights are used to computed tissue-specific gene set weights for all gene sets defined in **G**.

Possible variations and extensions of this pipeline are discussed in Section 4.2 below.

#### *2.2.1* Computation of tissue-specific gene weights

To compute tissue-specific gene weights, we use both mRNA and protein evidence from the HPA. Specifically, the weight $w_{i,t}^{g}$ for gene *i* in tissue *t* is computed as follows:

(1) $$w_{i,t}^{g}=e_{i,t}a_{i,t}$$

where:

- *e_i_*_,__*t*_ represents the expression fold-change for gene *i* in tissue *t* relative to the mean expression among all tissues supported by the HPA. In this case, expression values are taken from the HPA RNA-seq data in units of fragments per kilobase of transcript per million fragments mapped. If an RNA-seq measurement is missing for gene *i* in tissue *t*, *e_i_*_,__*t*_ is set to 0, i.e. we assume the gene is not expressed in tissue *t*.
- *a_i_*_,__*t*_ represents an indicator of gene activity based on IHC. Specifically, *a_i_*_,__*t*_ is set to 0 if the protein for gene *i* was not detected by the HPA IHC analysis in tissue type *t* and is set to 1 if the protein was detected at a ‘Low’ or greater level. If an IHC value is missing for gene *i* in tissue *t*, *a_i_*_,__*t*_ is set to 1, i.e. the overall tissue-specific gene weight is determined by just the RNA data if IHC measurements are missing.

Formula (1) results in a tissue-specific gene weight that requires evidence at both the protein and RNA level to generate a non-zero value. If both forms of evidence are available, the magnitude of the weight is set to the fold-change in expression of the gene in the target tissue relative to the mean in all tissues.

#### *2.2.2* Computation of tissue-specific gene set weights

The weight $w_{j,t}^{s}$ for gene set *j* and tissue type *t* is computed as the -log of the *P*-value from a competitive gene set test that compares the mean weight for tissue *t* of genes annotated to set *j* with the mean weight of all genes not in set *j*. Let **m** represent the genes annotated to set *j*, i.e. $m=\{i\in 1\ldots p\;\text{and}\;G_{j,i}=1\}$, let $m_{c}$ be the complement set, i.e. $m_{c}=\{i\in 1\ldots p\;\text{and}\;G_{j,i}=0\}$ and let |m| and $\left|m_{c}\right|$ represent the sizes of these sets. The competitive gene set test performed using a one-sided, two-sample *t*-test that evaluates the following null and alternative hypotheses:

(2) $$\begin{array}{c} H_{0}:1/|m|\sum_{i\in m}w_{i,t}^{g}=1/|m_{c}|\sum_{i\in m_{c}}w_{i,t}^{g}\\ H_{A}:1/|m|\sum_{i\in m}w_{i,t}^{g}>1/|m_{c}|\sum_{i\in m_{c}}w_{i,t}^{g} \end{array}$$

This form of test is very similar to the competitive gene set test implemented by the *geneSetTest* method in the R limma package (Ritchie *et al.*, 2015). The weight for gene set *j* and tissue type *t* can therefore be formally defined as:

(3) $$w_{j,t}^{s}=-\text{log}(P{\mathrm{val}}_{j,t})$$

where $p\mathrm{va}l_{j,t}$ is the *P*-value from this *t*-test. It is important to note that this form of competitive test assumes independence of the gene-level weights or, more precisely, a similar dependence structure among all weights. If all gene-level weights do not share a similar dependency structure, the magnitude of *w^s^* will be a function of both the difference in mean gene-level weights and the difference in correlation among gene-level weights (Wu and Smyth, 2012), i.e. large *w^s^* will be generated for gene sets whose member weights are much larger than average or are more highly correlated than average. For our applications of *w^s^*, this property is desirable. For additional discussion of this topic and the scenarios when it is acceptable to ignore inter-gene correlation during gene set testing, please see the CAMERA paper (Wu and Smyth, 2012) and the documentation for the *camera* function in the *limma* R package (Ritchie *et al.*, 2015).

## 3 Results

### 3.1 Catalog of tissue-specific gene set weights

Using the analytical approach detailed in Section 2, we have generated a public catalog of tissue-specific weights for 13 distinct MSigDB version 6.0 gene set collections (Table 1) and 37 human tissue types from the HPA (Table 2). This catalog can be accessed at http://www.dartmouth.edu/∼hrfrost/TissueSpecificGeneSets. This web site also includes R (R Core Team, 2016) code that implements our approach and can be used to generate tissue-specific weights for any desired gene set.

**Table 2.** Analyzed HPA tissue types

Adipose tissue	Gallbladder	Seminal vesicle
Adrenal gland	**Heart muscle**	**Skeletal muscle**
Appendix	Kidney	Skin
Bone marrow	**Liver**	**Small intestine**
Breast	**Lung**	Smooth muscle
**Cerebral cortex**	Lymph node	Spleen
Cervix, uterine	Ovary	Stomach
**Colon**	**Pancreas**	Testis
Duodenum	Parathyroid gland	Thyroid gland
Endometrium	Placenta	Tonsil
Epididymis	Prostate	Urinary bladder
**Esophagus**	Rectum	
Fallopian tube	Salivary gland	

*Note*: The 37 HPA tissue types for which tissue-specific gene sets weights were computed. The tissue types in bold were used to generate the analysis results in Section 3.

**Table 3.** MSigDB genes sets specific to adipose tissue, heart muscle and liver

	C2.CP		C5.BP	
Tissue	Gene set	Weight	Gene set	Weight
Adipose tissue	REACTOME_HORMONE_SENSITIVE_ LIPASE_HSL_MEDIATED_TRI…	133	GO_REGULATION_OF_ LIPID_STORAGE	135
	REACTOME_TRANSCRIPTIONAL_ REGULATION_OF_WHITE_ADIPO…	102	GO_REGULATION_OF_ SEQUESTERING_OF_TRIGLYCERIDE	125
	KEGG_PPAR_SIGNALING_PATHWAY	93	GO_LIPID_STORAGE	102
	REACTOME_LIPID_DIGESTION_ MOBILIZATION_AND_TRANSPOR…	59	GO_LOW_DENSITY_ LIPOPROTEIN_PARTICLE_CLEARANCE	85
	REACTOME_TRIGLYCERIDE_ BIOSYNTHESIS	48	GO_BROWN_FAT_CELL_ DIFFERENTIATION	83
	KEGG_ADIPOCYTOKINE_SIGNALING_ PATHWAY	42	GO_POSITIVE_REGULATION_ OF_LIPID_STORAGE	81
	REACTOME_FATTY_ACID_ TRIACYLGLYCEROL_AND_KETONE_BOD…	32	GO_REGULATION_OF_LIPID_ CATABOLIC_PROCESS	79
	REACTOME_METABOLISM_OF_LIPIDS_ AND_LIPOPROTEINS	29	GO_TRIGLYCERIDE_CATABOLIC_ PROCESS	75
	NABA_ECM_GLYCOPROTEINS	23	GO_REGULATION_OF_LIPID_METABOLIC_PROCESS	72
	BIOCARTA_LEPTIN_PATHWAY	23	GO_NEGATIVE_REGULATION_ OF_LIPID_STORAGE	68
Heart muscle	REACTOME_STRIATED_MUSCLE_CONTRACTION	304	GO_HEART_PROCESS	565
	KEGG_DILATED_CARDIOMYOPATHY	203	GO_STRIATED_MUSCLE_CONTRACTION	557
	KEGG_HYPERTROPHIC_CARDIOMYOPATHY_HCM	196	GO_CARDIAC_MUSCLE_TISSUE_ MORPHOGENESIS	515
	KEGG_CARDIAC_MUSCLE_CONTRACTION	179	GO_CARDIAC_MUSCLE_TISSUE_ DEVELOPMENT	511
	REACTOME_MUSCLE_CONTRACTION	169	GO_MUSCLE_CONTRACTION	464
	BIOCARTA_ALK_PATHWAY	110	GO_MYOFIBRIL_ASSEMBLY	460
	REACTOME_TCA_CYCLE_AND_RESPIRATORY_ ELECTRON_TRANSP…	39	GO_MUSCLE_SYSTEM_PROCESS	459
	REACTOME_RESPIRATORY_ELECTRON_ TRANSPORT_ATP_SYNTHE…	36	GO_CARDIAC_CELL_DEVELOPMENT	434
	KEGG_PARKINSONS_DISEASE	36	GO_ACTIN_MEDIATED_CELL_CONTRACTION	410
	REACTOME_RESPIRATORY_ELECTRON_ TRANSPORT	32	GO_MUSCLE_ORGAN_MORPHOGENESIS	402
Liver	KEGG_RETINOL_METABOLISM	280	GO_EPOXYGENASE_P450_PATHWAY	247
	KEGG_DRUG_METABOLISM_ CYTOCHROME_P450	252	GO_DRUG_METABOLIC_PROCESS	244
	REACTOME_BIOLOGICAL_OXIDATIONS	215	GO_MONOCARBOXYLIC_ACID_ METABOLIC_PROCESS	234
	KEGG_COMPLEMENT_AND_COAGULATION_ CASCADES	212	GO_ORGANIC_ACID_ METABOLIC_PROCESS	234
	KEGG_METABOLISM_OF_XENOBIOTICS_BY_ CYTOCHROME_P450	201	GO_ACUTE_PHASE_RESPONSE	228
	REACTOME_BILE_ACID_AND_BILE_SALT_ METABOLISM	191	GO_STEROID_METABOLIC_PROCESS	224
	REACTOME_PHASE1_FUNCTIONALIZATION_ OF_COMPOUNDS	187	GO_SMALL_MOLECULE_ METABOLIC_PROCESS	192
	REACTOME_XENOBIOTICS	185	GO_EXOGENOUS_DRUG_ CATABOLIC_PROCESS	183
	REACTOME_RECYCLING_OF_BILE_ACIDS_ AND_SALTS	155	GO_PROTEIN_ACTIVATION_CASCADE	177
	REACTOME_CYTOCHROME_P450_ARRANGED_ BY_SUBSTRATE_TYP…	152	GO_BILE_ACID_METABOLIC_PROCESS	177

*Note*: 10 MSigDB gene sets from the canonical pathways (C2.CP) and GO biological process (C5.BP) collections with the largest tissue-specific weights for adipose tissue, heart muscle and liver.

### 3.2 Characterization of human tissues

The tissue-specific gene set weights can be directly used to functionally characterize the associated human tissue. Specifically, the gene sets within a desired collection, e.g. MSigDB C2.CP, can be rank ordered according to the weight assigned to each set for a given tissue according to the procedure outlined in Section 2. The sets with the largest weights are expected to capture the primary biological processes active within that tissue. This procedure also enables the qualitative evaluation of the weights, i.e. do the gene sets with large weights accurately reflect the known features of the target tissue? To demonstrate this application, we analyzed the top-ranked MSigDB curated canonical pathways (C2.CP) and GO biological process (C5.BP) gene sets for adipose tissue, heart muscle and liver. As shown in Table 2, the top-ranked C2.CP and C5.BP gene sets accurately capture known biological properties of the associated tissues, e.g. lipid-related pathways for adipose tissue, cardiac-related pathways for heart muscle and metabolic pathways for liver. Similar results for the other 34 supported tissues can be found at http://www.dartmouth.edu/∼hrfrost/TissueSpecificGeneSets.

### 3.3 Multi-tissue analysis for systemic diseases

Characterization of a single tissue using the gene set weights can be extended to the analysis of a group of tissues, e.g. all human tissues impacted by a given systemic disease. For such a multi-tissue analysis, the gene sets within a collection can be rank ordered according to a multi-tissue weight calculated from the tissue-specific weights. Although numerous multi-tissue weights are possible (e.g. mean weight, median, etc.), we have found the minimum weight to be most effective for identifying biological processes associated with systemic diseases. If the set of analyzed tissues is represented by **t**, we compute the multi-tissue gene set weight for gene set *j*, $w_{j,t}^{m}$, as:

(4) $$w_{j,t}^{m}=\underset{t\in t}{\min}w_{j,t}^{s}$$

This form of multi-tissue weight highly ranks gene sets with at least a basic level of activity in all analyzed tissues. To demonstrate this approach to multi-tissue analysis, we analyzed the MSigDB C2.CP and C5.BP collections for four tissues that comprise the ‘dysharmonious quartet’ (Defronzo, 2009) of type II diabetes (T2D): adipose tissue, liver, pancreas and skeletal muscle. As seen in Table 4, this approach correctly captures processes with a known T2D association, e.g. various pathways relating to carbohydrate metabolism, insulin signaling and the very specific KEGG_TYPE_II_DIABETES_MELLITUS pathway.

**Table 4.** Multi-tissue analysis for T2D

C2.CP		C5.BP	
Gene set	Minimum	Gene set	Minimum
	weight		weight
REACTOME_METABOLISM_OF_CARBOHYDRATES	2	GO_GLUCOSE_METABOLIC_PROCESS	7.7
REACTOME_AMINO_ACID_SYNTHESIS_AND_ INTERCONVERSION_…	1.1	GO_HEXOSE_METABOLIC_PROCESS	6.5
KEGG_TYPE_II_DIABETES_MELLITUS	1.1	GO_NEGATIVE_REGULATION_OF_ CARBOHYDRATE_METABOLIC_P…	5.4
KEGG_INSULIN_SIGNALING_PATHWAY	1	GO_MONOSACCHARIDE_ BIOSYNTHETIC_PROCESS	5.4
KEGG_ALANINE_ASPARTATE_AND_GLUTAMATE_ METABOLISM	0.92	GO_CARBOHYDRATE_METABOLIC_ PROCESS	4.7
REACTOME_MITOCHONDRIAL_FATTY_ACID_ BETA_OXIDATION	0.69	GO_MONOSACCHARIDE_METABOLIC_ PROCESS	4.7
REACTOME_BRANCHED_CHAIN_AMINO_ ACID_CATABOLISM	0.65	GO_SMALL_MOLECULE_METABOLIC_ PROCESS	4.2
KEGG_PROXIMAL_TUBULE_BICARBONATE_ RECLAMATION	0.64	GO_REGULATION_OF_CARBOHYDRATE_ METABOLIC_PROCESS	3.7
BIOCARTA_SARS_PATHWAY	0.64	GO_REGULATION_OF_GLUCOSE_ METABOLIC_PROCESS	3.3
KEGG_VALINE_LEUCINE_AND_ISOLEUCINE_ DEGRADATION	0.62	GO_SMALL_MOLECULE_BIOSYNTHETIC_ PROCESS	2.9

*Note*: The 10 MSigDB gene sets from the curated canonical (C2.CP) and GO biological process (C5.BP) collections that have the largest minimum tissue-specific weight across four tissues significantly impacted by T2D (adipose tissue, liver, pancreas and skeletal muscle).

### 3.4 Tissue-specific gene set testing

The tissue-specific gene set weights can also be used to improve the performance of standard gene set testing. Specifically, the tissue-specific weights *w^s^* can be used to increase gene set testing statistical power via hypothesis or *P*-value weighting (Genovese *et al.*, 2006; Ignatiadis *et al.*, 2016). A key challenge encountered with gene set testing is the significant penalty on power caused by MHC (Frost *et al.*, 2015). This can be especially problematic when the analysis is performed using large gene set collections that contain thousands of sets. In hypothesis or *P*-value weighting, the unadjusted *P*-values from the family of tested hypotheses are modified by weights that reflect the prior likelihood that the alternative hypothesis is true. As detailed in Genovese *et al.* (Genovese *et al.*, 2006), the Benjamini and Hochberg (BH; Benjamini and Hochberg, 1995) method provides valid FDR control when applied to weighted *P*-values (i.e. weighted FDR or wFDR) as long as two key requirements are met: (i) the average weight is 1 and (ii) the weights are independent of the *P*-values under *H*_0_. In order to improve statistical power, the weights must additionally be inversely associated with the *P*-values under *H_A_*, i.e. the weights have to correctly prioritize true discoveries. In our application, the tissue-specific weights are completely independent of the data under analysis, which insures independence under *H*_0_. Note that it is also possible to ensure independence under *H*_0_ using weights computed from the analyzed data [e.g. the approach of Ignatiadis *et al.* (Ignatiadis *et al.*, 2016)]. To meet the requirement that the weights sum to 1, the tissue-specific gene set weights *w^s^* were standardized as:

(5) $$w_{j,t}^{s*}=\frac{w_{j,t}^{s}}{1/g\sum_{i=1}^{g}w_{i,t}^{s}}$$

If the *P*-value for gene set *j* from gene set testing is *P*val_*j*_, these standardized weights can then be used to generate weighted *P*-values, $pval_{j}^{*}$, as:

(6) $$pval_{j}^{*}=\frac{pval_{j}}{w_{j,t}^{s*}}$$

Given these weighted *P*-values, the wFDR *q*-values can be computed using the standard BH method applied to $pval_{j}^{*}$ instead of *P*val_*j*_. It is important to note that this procedure can be used with any desired gene set testing method as long the method performs hypothesis testing and therefore generates *P*-values.

Ensuring that the tissue-specific weights have an inverse association with gene set testing *P*-values under *H_A_* is more challenging and will not hold under all experimental conditions. In particular, we believe that this *P*-value weighting scheme will be most effective in two primary scenarios:

- When the goal of gene set testing is to identify dysregulation of gene sets that play a biologically important role in the target tissue, i.e. are active under normal conditions and are specific to the tissue. Since gene sets with large weights are more likely than gene sets with small weights to reflect processes specific to the target tissue and active under normal conditions, *P*-value weighting should improve statistical power.
- When the dependent variable in gene set testing is expected to show the most significant association with processes that are normally active and specific to the tissue under analysis. An example of such a dependent variable would be an intervention that impacts the function of active processes in the target tissue rather than one that activates normally inactive processes. Because gene sets with large weights are more likely to reflect normally active and tissue-specific processes than gene sets with low weights, *P*-value weighting should improve statistical power in this scenario.

It is important to also note scenarios where the proposed *P*-value weighting is unlikely to work well, i.e. cases where the weights are not associated with gene set testing *P*-values under the desired *H_A_*. These problematic scenarios (and potential alternate approaches) include:

- When the phenotype is associated with gene sets that are not normally active in the target tissue. In this case, the *P*-values for significant gene sets will be down-weighted with an associated loss of power. One potential approach for this scenario involves filtering the gene set collection to remove sets with a tissue-specific weight above a given threshold.
- When the phenotype is associated with gene sets whose members are ubiquitously expressed in all tissues, e.g. housekeeping processes. In this case, *P*-value weighting will rank tissue-specific processes above the ubiquitous processes with an associated loss in power. A potential approach in this scenario involves the use of a tissue-agnostic gene set weight rather than a tissue-specific weight. Such a weight could be based on the proportion of gene set members that are ubiquitously expressed in all tissues.
- When the phenotype is associated with gene sets that are active in tissues other than the tissue under investigation. In this case, the proposed *P*-value weighting will prioritize the wrong group of gene sets with an associated loss in power. If the appropriate tissue is known, researchers can address this scenario by simply using the appropriate weights. If the correct tissue is not known a priori, then a comparative analysis of results using weights for a range of tissues might prove effective.
- When certain gene sets have very large tissue-specific weights relative to other sets in the collection. In this case, *P*-values that are nominally insignificant can generate significant *q*-values via wFDR analysis. To address this case, researchers could discretize the gene set weights, i.e. filter the collection prior to hypothesis testing.

To demonstrate the effectiveness of this approach, we performed gene set testing using the MSigDB C2.CP collection on normalized gene expression data from version V6p of the GTEx (GTEx Consortium, 2015) for 10 tissue types relative to 10 phenotypes shown in Table 5 for total of 100 distinct tissue/phenotype combinations. Gene set testing was performed using the competitive method CAMERA, as implemented by the *camera* method in the R limma package and using default settings (Wu and Smyth, 2012). For each of these tissues, the gene set testing results using FDR control via the BH method was compared against the results from wFDR using the weights defined in Equation (5).

**Table 5.** Tissue-specific gene set testing results

	Age	BMI	Cerebrovascular	COPD	Depression	Gender	Heart	Hyper	Liver	T2D
			Disease				Disease	-Tension	Disease	
Adipose tissue (subcutaneous)		4/7		3/6		0/1	0/1		2/2	**2/1**
Cerebral cortex			0/3		0/2					
Colon (transverse)	**4/1**	**3/1**		**17/1**					**4/0**	**3/2**
Esophagus mucosa	**8/7**	1/1		**1/0**			**11/3**	**6/4**	**1/0**	**1/0**
Heart (left ventricle)	24/46	**2/0**		**2/0**	**9/8**	**3/0**	**3/0**		**11/8**	
Liver	**17/3**	**8/0**	**24/5**	**20/1**	**10/0**	**12/6**	**9/0**		**9/0**	**10/0**
Lung								0/1		
Pancreas					0/1	**2/1**				
Skeletal muscle		**1/0**	**3/0**		**4/0**	**1/0**		**5/4**		**2/1**
Small intestine (terminal ileum)		**10/0**	**11/3**	**21/8**		**8/0**	**8/0**		**8/0**	**9/0**

*Note*: Number of discoveries at an FDR *q*-value ≤ 0.2 (weighted discoveries/unweighted discoveries) from a gene set testing analysis of GTEx gene expression data from 10 different tissues relative to 10 different phenotypes using the MSigDB v6.0 C2.CP collection. Tissue and phenotype combinations with no discoveries are blank. If the weighted analysis yielded more discoveries than the unweighted analysis, the cell text is bold.

As shown in Table 5, the wFDR analysis yields more findings at a *q*-value ≤ 0.2 for 41 of the 52 tissue/phenotype combinations with at least one significant finding. Overall, the use of tissue-specific *P*-values weights generated a total of 337 discoveries versus just 139 for the unweighted analysis. Details for all significant gene set findings can be found at http://www.dartmouth.edu/∼hrfrost/TissueSpecificGeneSets. Importantly, the additional gene set findings generated by the wFDR analysis are, in general, biologically plausible for the associated phenotype with significant nominal *P*-values. As an illustrative example, Table 6 lists the 10 gene set findings generated by the wFDR analysis for liver relative to T2D status with a sample of references supporting association of the gene set with T2D.

**Table 6.** Significant pathways in GTEx liver relative to T2D

Gene set	Weight	P-value	FDR	wFDR	Support for TD2 association
KEGG_SNARE_INTERACTIONS_IN_VESICULAR_TRANSPORT	3.7	0.00047	0.62	0.08	(Zhu *et al.*, 2017)
REACTOME_PROTEOLYTIC_CLEAVAGE_OF_SNARE_COMPLEX_PROTEINS	23	0.0052	0.69	0.08	(Zhu *et al.*, 2017)
REACTOME_FGFR1_LIGAND_BINDING_AND_ACTIVATION	11	0.0025	0.62	0.08	(Wu *et al.*, 2011)
REACTOME_AQUAPORIN_MEDIATED_TRANSPORT	26	0.0063	0.76	0.08	(Lloyd *et al.*, 2005)
REACTOME_SIGNALING_BY_ACTIVATED_POINT_MUTANTS_OF_FGFR1	10	0.0033	0.63	0.085	(Wu *et al.*, 2011)
REACTOME_G_BETA_GAMMA_SIGNALLING_THROUGH_PI3KGAMMA	10	0.004	0.67	0.089	(Azzi *et al.*, 2017)
PID_HDAC_CLASSII_PATHWAY	66	0.048	1	0.14	(Ye, 2013)
REACTOME_PI3K_CASCADE	12	0.01	0.87	0.14	(Boucher *et al.*, 2014)
REACTOME_MEIOTIC_SYNAPSIS	59	0.057	1	0.14	(Kim *et al.*, 2007)
REACTOME_FACILITATIVE_NA_INDEPENDENT_GLUCOSE_TRANSPORTERS	47	0.056	1	0.16	(Baud *et al.*, 2016)

*Note*: Top 10 MSigDB canonical pathways whose gene expression values in GTEx liver samples are most significantly associated with T2D status.

## 4 Discussion

Gene set testing, or pathway analysis, is an effective and widely used hypothesis aggregation technique. By focusing on the collective effect of biologically meaningful groups of genomic variables, rather than just the marginal effect of individual genes, gene set testing methods can significantly improve statistical power, replication of results and biological interpretation. Despite the significant progress made building gene set collections and developing gene set testing methods, the practical utility of this technique is limited by challenges including annotation quality, statistical power and tissue specificity. Although the function and activity of many genes is tissue-specific, gene set testing is normally performed using tissue agnostic gene sets with no computational adjustments to account for the source tissue. This practice can significantly impact gene set testing accuracy whenever a mismatch exists between the experimental tissue and either the tissue used as evidence for an annotation or the tissue associated with the process or function represented by a gene set.

To address this challenge, we developed a bioinformatics approach for computing tissue-specific gene set weights using both RNA-seq and IHC evidence from the HPA regarding the tissue-specific activity of human protein-coding genes. This research represents an important advance in support for tissue-specific gene set analysis. Key contributions include:

- **A comprehensive repository of tissue-specific gene set weights.** The proposed method has been used to create a public repository of tissue-specific weights for 17 770 MSigDB gene sets representing a wide range of biological processes and experimental results. These weights were generated using evidence at both the RNA level (via RNA-seq) and protein level (via IHC) for 37 human tissue types profiled in the HPA. This repository can be accessed at
- http://www.dartmouth.edu/∼hrfrost/TissueSpecificGeneSets.
- **Software that can be used to compute tissue-specific weights for any gene set collection.** The repository of tissue-specific gene set weights includes an R implementation of the weight generation method. This software can be used by other researchers to generate tissue-specific weights for any desired collection of gene sets for any of the 37 supported HPA tissue types. This logic also supports a number of options that enable researchers to customize the weight generation algorithm (e.g. use either RNA or IHC evidence, modify the discretization of IHC evidence, etc.).
- **An approach for characterizing the biological features of individual human tissues.** As detailed in Section 3.2, the tissue-specific gene set weights provide a direct means for identifying the distinctive biological traits of specific human tissues. This information can be leveraged to help select the most appropriate tissue for a given investigation or to guide the analysis of experimental data generated in a specific tissue.
- **An approach for identifying processes common to a group of human tissues.** As detailed in Section 3.3, the tissue-specific gene set weights can be used to jointly profile multiple human tissue types. Use cases for this type of analysis include the study systemic diseases and investigation of environmental exposures impacting multiple tissues.
- **An approach for leveraging the tissue-specific weights to improve gene set testing performance.** As detailed in Section 3.4, the tissue-specific gene set weights can be used to improve the statistical power of gene set testing through a wFDR analysis. This technique can significantly improve the likelihood of identifying biologically valid gene set associations from experiments that generate high-dimensional genomic data.

### 4.1 Limitations

Although the initial results (as detailed in Section 3) are encouraging and clearly demonstrate the validity and utility of the computed gene set weights, there are some important limitations of our approach. In addition to the problematic scenarios identified in Section 3.4, these include:

- **Uncertainty regarding tissue-specific gene activity.** The HPA RNA and IHC measurements used to generate the gene set weights are estimates based on a finite number of samples and therefore only approximate the true population values. Additionally, these measurements reflect mRNA and protein abundance which may be imperfect proxies for the true functional activity of a protein.
- **Process used to compute gene-set weights.** The method used to compute the gene set weights involves a number of approximations and simplifying assumptions that may impact the quality and biological validity of the weights. These include the model used to combine RNA and IHC evidence, the discretization of the IHC data and the assumption that the protein has low activity if IHC measurements are missing for that tissue.
- **Application to neoplastic or morphologically abnormal tissue.** Because the HPA measurements were made on non-neoplastic and morphologically normal tissue samples, the derived gene weights may provide a poor reflection of gene expression and protein activity in neoplastic or morphologically abnormal tissues.

### 4.2 Future directions

Possible extensions or refinements of this work include addressing the problematic scenarios detailed in Section 3.4, the use of information regarding tissue-specific gene activity to filter gene set annotations or weight those annotations during gene set testing, modifications to account for the exact level of protein activity reported in the HPA IHC data, integration of other sources of tissue-specific gene activity and the extension to cell lines, model organisms and neoplastic tissue.

## Funding

This work has been supported by National Institutes of Health grant K01LM012426.

*Conflict of Interest*: none declared.
